# Behavior of NiTiNb SMA wires under recovery stress or prestressing

**DOI:** 10.1186/1556-276X-7-66

**Published:** 2012-01-05

**Authors:** Eunsoo Choi, Tae-hyun Nam, Young-Soo Chung, Yeon-Wook Kim, Seung-yong Lee

**Affiliations:** 1 Department of Civil Engineering, Hongik University, Seoul, 121-791, South Korea; 2 Department of Metal and Material Engineering, GyeongSang National University, Jinju, 660-701, South Korea; 3 Department of Civil Engineering, Chung-Ang University, Seoul, 156-756, South Korea; 4 Department of Advanced Materials Engineering, Keimyung University, Daegu, 704-701, South Korea; 5 Department of Civil Engineering, Chungju National University, Chungju, 380-702, South Korea

**Keywords:** shape memory alloys, recovery stress, residual stress, NiTiNb, confinement

## Abstract

The recovery stress of martensitic shape-memory alloy [SMA] wires can be used to confine concrete, and the confining effectiveness of the SMA wires was previously proved through experimental tests. However, the behavior of SMA wires under recovery stress has not been seriously investigated. Thus, this study conducted a series of tests of NiTiNb martensitic SMA wires under recovery stress with varying degrees of prestrain on the wires and compared the behavior under recovery stress with that under prestressing of the wires. The remaining stress was reduced by the procedure of additional strain loading and unloading. More additional strains reduced more remaining stresses. When the SMA wires were heated up to the transformation temperature under prestress, the stress on the wires increased due to the state transformation. Furthermore, the stress decreased with a decreasing temperature of the wires down to room temperature. The stress of the NiTiNb wires was higher than the prestress, and the developed stress seemed to depend on the composition of the SMAs. When an additional strain was subsequently loaded and unloaded on the prestressed SMA wires, the remaining stress decreased. Finally, the remaining stress becomes zero when loading and unloading a specific large strain.

## Introduction

The shape-memory effect produces recovery stress when deformed shape-memory alloy [SMA] wires are heated over *A*_f_, where the transformation to austenite is completed, with restraining deformation [1]. The developed or remaining recovery stress depends on the temperature of the wire and becomes zero when the temperature decreases to *M*_s_, where the martensite starts. Furthermore, the recovery and residual stresses depend on the alloy types, such as NiTi or NiTiNb, and the temperature window of the SMA alloys [2,3]. The recovery stress can be used to provide external confinement for reinforced concrete columns [3] or prestress in reinforced concrete beams [4]. Several previous studies showed that SMA wires were very effective in providing external confinement for concrete [5,6]. As an external jacket, SMA wire jackets increased the peak strength of concrete and the ductility of reinforced concrete columns. In this case, the shape-memory effect of SMAs was involved, and the SMA wires were tensioned under residual stress due to the expansion of the concrete. With a beam, the recovery stress provided compressive prestress on the concrete of the beam [7]. The SMA wires or bars in both cases were tensioned cyclically due to loading and unloading of live loads. Thus, the wire or bars were exposed to a hysteretic behavior under recovery stress.

No experimental tests or analysis of the behavior of SMA wires under recovery stress have been conducted. Thus, we conducted cyclic tensile tests of SMA wires under recovery stress and analyzed the results. This study also investigated the hysteretic behavior of SMA wires under prestress.

## Cyclic behavior under recovery stress

### SMA wires

**Figure 1:**
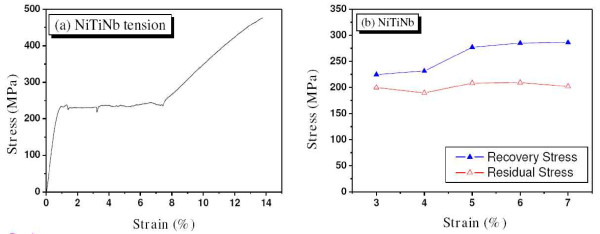
**The NiTiNb SMA wire**. (**a**) Stress-strain curve. (**b**) Recovery and residual stresses with variation of prestrain.

This study used SMA wires of Ni_47.45_-Ti_37.86_-Nb_14.69 _with a 1.0-mm diameter. The alloy was prepared by high-frequency vacuum induction melting and then hot-rolled into wires with a diameter of 1.075 mm at 850°C. The hot-rolled wires were deformed into a wire with a diameter of 1.0 mm by cold-drawing without intermediate annealing. The process induced a prestrain of approximately 7% in the SMA wires. The temperature windows of the NiTiNb alloy are shown in Table 1. The *M*_s _of -17.59°C was less than -10°C, and the *A*_s _of 104.91°C was larger than 40°C, and thus, the temperature condition perfectly satisfied the requirement for civil structures mentioned in a previous study [3]. Therefore, the NiTiNb SMA wires can be stored safely under an ambient temperature and retain residual stress under cool temperatures, such as -10°C. Figure 1a shows the stress-strain curve of the SMA wires with monotonic loading. For the NiTiNb SMA, the transformation started at a 0.93% strain with 231.6 MPa. The stress-induced martensite hardening began at a 7.5% strain with 242.2 MPa.

**Table 1:** Temperature windows of NiTiNb alloy

Alloy	*M*_s_ (°C)	*M*_f_ (°C)	*A*_s_ (°C)	*A*_f_ (°C)	*A*_s _- *M*_s_ (°C)
NiTiNb	-17.59	-74.29	104.91	139.18	122.5

### Test procedure

When a prestrained martensitic SMA wire with constraining deformation is heated over a temperature of *A*_s_, recovery stress develops in the wire. If the temperature is cooled to room temperature, the recovery stress is reduced, and the remaining stress is called the residual stress. This study conducted cyclic tensile loading tests of the SMA wires under residual stress. To produce the residual stress, the SMA wires were elongated with a prestrain of 3% to 7%, increased by 1%, and unloaded. Next, the wires were heated to 200°C and then cooled to 25°C. The recovery and residual stresses that developed are shown in Figure 1b. The recovery and residual stresses were almost stable beyond a 5% prestrain with 286 MPa and at a 7% prestrain with 202 MPa, respectively. Finally, the wires under residual stress were loaded with cyclic loadings: at first, the wire was elongated up to a 0.2% strain additionally and unloaded to the original residual strain, and then, the wire was reloaded up to a 0.4% strain and unloaded. The cyclic loading assigned was continuously increasing by a 0.2% strain additionally until all the residual stresses disappeared.

### Test results of NiTiNb SMA wires

**Figure 2:**
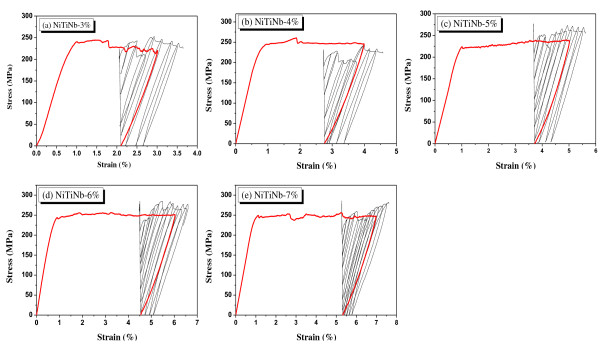
**Cyclic curves of NiTiNb SMA wires under residual stress**.

The loading for prestrain and unloading curve and the subsequent hysteretic curves in the NiTiNb SMA wires are shown in Figure 2. The reloading slopes from the initial residual stress appeared to be equal to the slopes of the unloading stiffness from the prestrains. The reloading curves crossed the plateau-stress line, and the maximum stress of the reloading seemed to be equal to the plateau stress: Figure 2e shows this almost perfectly. The residual stress decreased with an increasing reloading strain when the wire was unloaded. When the reloading strain reached the prestrain, the residual stress became zero with subsequent unloading. The reloading beyond the prestrain and the subsequent unloading remained a residual strain.

**Figure 3:**
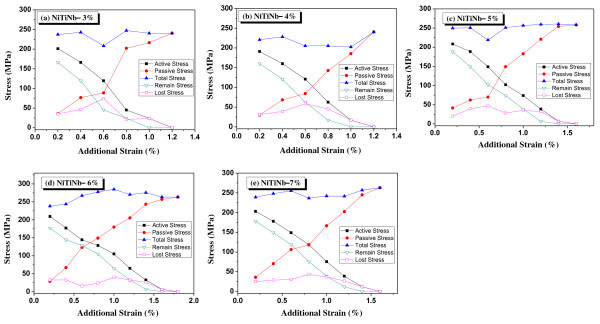
**Analysis of cyclic curves according to additional strain under residual stress for NiTiNb SMA wires**.

Figure 3 shows the analysis of each hysteretic curve according to the additional strains. In the figure, the total stress was the summation of the active and passive stresses. The active stress was the remaining residual stress that provided active confinement when the additional strain began.

**Figure 4:**
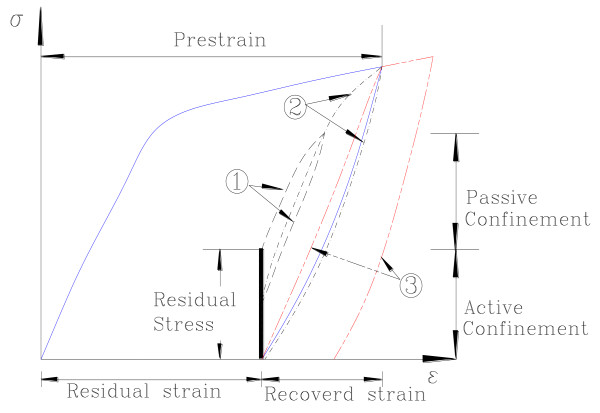
**Schematic cyclic behavior of an SMA wire under residual stress**.

The passive stress developed because of the additional strain, and the remaining stress was measured when the unloading went back to the original residual strain. Thus, the previous remaining stress acted as the active stress for the next additional strain procedure. Figure 4 shows the active and the passive confining stresses at the first reloading case as in ① in the figure. The last lost stress was the amount of stress reduction due to a reloading-and-unloading cycle. Thus, the summation of the remaining and lost stresses was equal to the active stress. The total stress showed a flat trend; this means that the first additional strain reached the plateau-stress line. When the remaining stress becomes zero, all the residual stresses disappeared. The additional strains at zero remaining stress ranged from 1.0% to 1.4%; the strains almost corresponded to the recovered strains in Figure 2. The NiTiNb SMA wires acted like a viscoelastic spring in the range from the initial residual strain to the original prestrain since no additional strain developed due to cyclic loading in that range. Choi et al. [3] called the range an available range which was equal to the recovered strain. For the application of confining concrete by SMA wires, the range exceeding the available range may not be used because the wire in that range becomes longer than the perimeter of a cylinder or a column wrapped by the wire after unloading, and thus, may not provide any confinement on concrete.

## Discussion of results

Choi et al. [3] explained the hysteretic behavior of an SMA wire under residual stress as shown in Figure 4. They indicated that the reloading curve passed the prestrain point (② in Figure 4) and the residual stress became zero with unloading from the prestrain. When the reloading strain exceeded the prestrain, the residual strain remained with unloading as in ③. However, based on the above observations, the reloading curves did not pass the prestrain point. Therefore, the behavior in Figure 4 seems to be a special case: the reloading curve appears to cross the plateau-stress line, the prestrian point, or the unloading line from the prestrain. The factors that determine the reloading path would be the amount of the initial residual stress, the types of SMA alloys, and so on: a further study is required to determine all the related factors. Thus, the assumption suggested by Choi et al. [3] was partially correct.

### Cyclic behavior under prestressing

**Figure 5:**
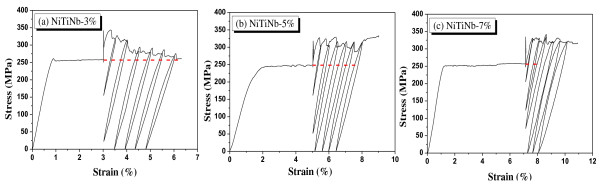
**Hysteretic behavior of NiTiNb and NiTi SMA wires under prestress**.

The NiTiNb SMA wires were prestrained up to 3%, 5%, or 7% and had constrained deformation. The wires were then heated to 200°C and cooled to room temperature. This process produced recovery and residual stresses as shown in Figure 5. After that, the wires were elongated cyclically with increasing strains; the maximum stress was larger than the plateau stress developed during the monotonic loading. The maximum developed stress due to reloading was larger than the plateau stress: for a 7% prestrain, the maximum developed stress was approximately 325 MPa, which was larger by 28.5% than the plateau stress of 253 MPa. Therefore, the procedure can provide more confining pressures or prestresses than in the case of the residual stress in Figure 2.

## Conclusions

This study investigated the hysteretic behavior of NiTiNb SMA wires under residual stress experimentally and corrected the previous assumption of the behavior. The reloading curve crossed the plateau-stress line or the unloading line. In general, it appears that the initial residual stress is close to the plateau stress, and then, the reloading curve crosses the plateau-stress line. However, the initial residual stress is much lower than the plateau stress, and then, the reloading curve crosses the unloading line. For the first case, the available range was equal to the recovered strain; however, for the second case, the range was smaller than the recovered strain. Therefore, SMA wires that show the behavior of the first case are appropriate to apply in confining concrete. This study also investigated the behavior of SMA wires with prestress. The NiTiNb SMA wire under prestress was heated, and then, recovery and residual stresses developed. Under that condition, the wire showed more stresses than the plateau stress. Through the behavior of NiTiNb SMA wires under residual stress and under prestressing, the *M*_s _of SMA wires for a safe application in confining concrete should be lower than the lowest air temperature.

## Competing interests

The authors declare that they have no competing interests.

## Authors' contributions

EC coordinated this study and carried out the analysis of the data. T-HN and Y-SC participated in the tensile tests of the SMA wires, and Y-WK conducted the material test of the SMAs to measure the temperature windows and components of the SMAs. S-YL participated in manufacturing the SMA wires. All authors have read and approved the final manuscript.
